# Comprehensive use of cardiac computed tomography to guide left ventricular lead placement in cardiac resynchronization therapy

**DOI:** 10.1016/j.hrthm.2017.04.041

**Published:** 2017-09

**Authors:** Jonathan M. Behar, Ronak Rajani, Amir Pourmorteza, Rebecca Preston, Orod Razeghi, Steve Niederer, Shaumik Adhya, Simon Claridge, Tom Jackson, Ben Sieniewicz, Justin Gould, Gerry Carr-White, Reza Razavi, Elliot McVeigh, Christopher Aldo Rinaldi

**Affiliations:** ∗ Department of Imaging Sciences & Biomedical Engineering, King’s College London, London, United Kingdom; † Cardiology Department, Guy’s and St Thomas’ NHS Foundation Trust, London, United Kingdom; ‡ Department of Radiology and Imaging Sciences, National Institutes of Health Clinical Center, Bethesda, Maryland; § Departments of Bioengineering, Medicine, and Radiology, University of California San Diego, La Jolla, California

**Keywords:** Cardiac computed tomography, Cardiac resynchronization therapy, CT guided intervention, Dyssynchrony, Myocardial fibrosis

## Abstract

**Background:**

Optimal lead positioning is an important determinant of cardiac resynchronization therapy (CRT) response.

**Objective:**

The purpose of this study was to evaluate cardiac computed tomography (CT) selection of the optimal epicardial vein for left ventricular (LV) lead placement by targeting regions of late mechanical activation and avoiding myocardial scar.

**Methods:**

Eighteen patients undergoing CRT upgrade with existing pacing systems underwent preimplant electrocardiogram-gated cardiac CT to assess wall thickness, hypoperfusion, late mechanical activation, and regions of myocardial scar by the derivation of the stretch quantifier for endocardial engraved zones (SQUEEZ) algorithm. Cardiac venous anatomy was mapped to individualized American Heart Association (AHA) bull’s-eye plots to identify the optimal venous target and compared with acute hemodynamic response (AHR) in each coronary venous target using an LV pressure wire.

**Results:**

Fifteen data sets were evaluable. CT-SQUEEZ–derived targets produced a similar mean AHR compared with the best achievable AHR (20.4% ± 13.7% vs 24.9% ± 11.1%; *P* = .36). SQUEEZ-derived guidance produced a positive AHR in 92% of target segments, and pacing in a CT-SQUEEZ target vein produced a greater clinical response rate vs nontarget segments (90% vs 60%).

**Conclusion:**

Preprocedural CT-SQUEEZ–derived target selection may be a valuable tool to predict the optimal venous site for LV lead placement in patients undergoing CRT upgrade.

## Graphical abstract

**Figure:**
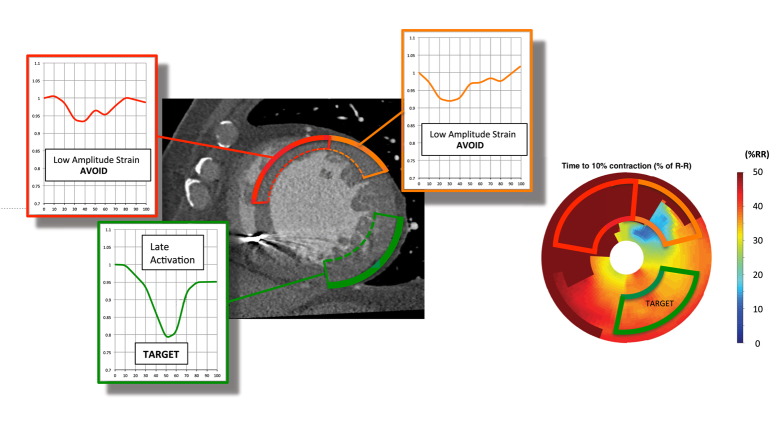

## Introduction

Patients with existing pacing systems, left ventricular (LV) systolic impairment, and a high proportion of right ventricular (RV) pacing benefit from cardiac resynchronization therapy (CRT).^1^ CRT nonresponse occurs because of suboptimal LV lead positioning in myocardial scar with persistent dyssynchrony.^2^, ^3^ Cardiac magnetic resonance (CMR) can guide LV lead placement by avoiding scar and targeting late mechanical activation (LMA)^4^; however, 28% of patients undergoing CRT have existing pacing systems unsuitable for CMR.^5^ Cardiac computed tomography (CT) has the potential to guide LV lead placement in patients with existing pacing systems.^6^ Rapid acquisition of 3-dimensional, isotropic, whole heart data sets with submillimeter spatial resolution can accurately delineate the coronary venous tree,^7^ noninvasively assess regional and global LV function,^8^ and detect regional hypoperfusion/myocardial scar.^9^ Recently, CT has evaluated regional and global LV dyssynchrony and areas of LMA by calculating the stretch of the endocardial surface throughout the cardiac cycle (stretch quantifier for endocardial engraved zones [SQUEEZ]).^10^ In patients with existing pacing systems undergoing CRT, we hypothesized that preprocedural cardiac CT-SQUEEZ by targeting areas of LMA and avoiding myocardial scar could guide LV lead placement through identification of the optimal venous target.

## Methods

The study complied with the Declaration of Helsinki, and the protocol was approved by the local ethics committee. Informed consent was obtained from each patient. Between September 2014 and July 2016, we prospectively recruited 18 patients with a preexisting pacemaker/implantable cardioverter-defibrillator, persistent heart failure symptoms on optimal medical therapy, LV ejection fraction <45%, and >50% RV pacing.

### Preassessment

Patients underwent clinical assessment (New York Heart Association score and Minnesota Living with Heart Failure questionnaire), 6-minute walk test, cardiopulmonary exercise test, and 2-dimensional transthoracic echocardiography. Ischemic cardiomyopathy (ICM) was defined by prior myocardial infarction, coronary angiography demonstrating severe coronary disease and subsequent revascularization, and/or CMR evidence of myocardial fibrosis. Absence of these features inferred non-ICM.

### Cardiac CT

Patients underwent cardiac CT using the Brilliance iCT 256-slice MDCT scanner (Philips Healthcare, Best, The Netherlands) before upgrade. Intravenous metoprolol was used to achieve a heart rate of <65 beats/min in sinus rhythm and <100 beats/min in atrial fibrillation. A total of 120 mL of intravenous contrast (Omnipaque, GE Healthcare, Princeton, NJ) was injected (5 mL/s) via a power injector into the antecubital vein. Descending aorta contrast-triggered (180 Hounsfield units [HU]), electrocardiogram (ECG)–gated scanning was performed with single breath-hold technique after a 10- to 12-second delay. Scanning parameters included a heart rate–dependent pitch of 0.2–0.45, a gantry rotation time of 270 ms, a tube voltage of 100 or 120 kVp depending on the patient’s body mass index, and a tube current of 125–300 mA depending on the thoracic circumference. A second single-phase ECG-gated scan was acquired 12 minutes after the initial contrast bolus for myocardial scar imaging; the tube voltage was reduced by 20 kVp while the tube current was increased proportionally to account for an increase in image noise.^11^ Initial retrospective ECG-gated scans were reconstructed in 10% phase increments throughout the cardiac cycle using iterative reconstruction, with 1-mm slice thickness, 0.5-mm slice increment, 250-mm field of view, 512 × 512 matrix, and an *Xres smooth* reconstruction kernel. Iterative reconstruction using the iDose4 algorithm (range 1–7) was used to reduce image noise and radiation dose. The cardiac CT scan was evaluated by an independent Society of Cardiovascular Computed Tomography Level III cardiac CT expert (R.R.). First-pass contrast-enhanced sequences were analyzed using standardized multiplanar reconstruction windows according to the AHA nomenclature for regional segmentation. End-diastolic myocardial wall thickness was evaluated reviewing each myocardial region in both short- and long-axis views. Areas of hypoperfusion were evaluated systematically with slice width thickness increased to 5 mm and display window and level settings adjusted to 100 and 200 HU, respectively. Abnormal perfusion was defined as myocardium exhibiting significantly reduced contrast distribution visually compared against attenuation of normal myocardium in each patient used as an internal reference. Where discrepancies existed, a 50 HU difference between normal and hypoperfused myocardium was used^12^, ^13^ along with consensus opinion from 2 individual experts in cardiac CT (AHA/ACC Level III experience, blinded to the clinical data).

We adopted a pragmatic approach for identifying regions of delayed enhancement as previously described.^11^ This qualitative identification of scar where the myocardium is brighter reflects the current lack of standard criteria in the literature mainly because the Hounsfield unit attenuation varies significantly between patients with no accepted value set as a cutoff for fibrosis. Where scar detection was difficult, surrogate markers of a wall thickness of <6 mm and regional hypoperfusion were used to infer scar.

### CT-derived SQUEEZ

**Figure 1:**
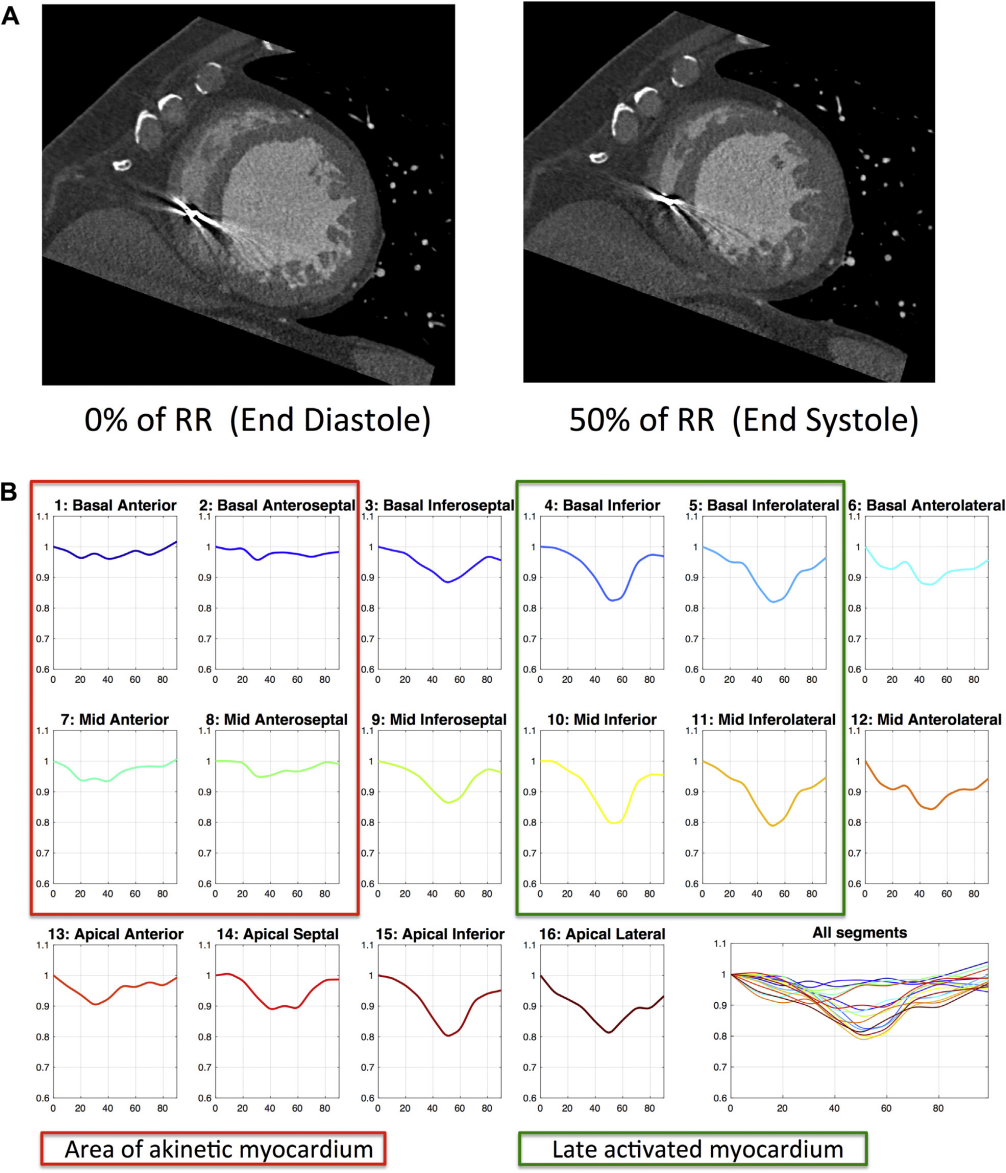
**A:** Computed tomography mid-ventricular short-axis images of the left ventricle at 2 time points in the R-R interval (0% = end diastole; 50% = end systole). Anterior and anteroseptal regions are akinetic and seen not to move throughout the cardiac cycle compared with the inferolateral segments that move inward by end systole. Inevitable beam hardening artifact from the existing pacing system noted in the right ventricle. **B:** Stretch quantifier for endocardial engraved zones values (y axis) vs cardiac cycle length (%) across 16 AHA segments demonstrate akinetic regions (red box) and late activating inferior/inferolateral walls (green box representing an ideal target for left ventricular lead placement).

High-contrast disparity between LV blood pool and myocardium permits the identification and tracking of finely engraved endocardial surface features. The SQUEEZ method uses these features to track endocardial material points over the heart cycle to calculate regional cardiac function using the following formula:

$$\text{SQUEEZ}\left(\text{v},\text{t}\right)\approx \frac{\sqrt{\text{A}}\left(\text{v},\text{t}\right)}{\sqrt{\text{A}}\left(\text{v},0\right)}$$

where A(v, 0) is the area of the small triangular patch (v) on the endocardial mesh at end diastole and A(v, t) is the area of the same patch at time t. The SQUEEZ metric is calculated for each of the triangular patches across the endocardium through cardiac phases. Thus, a high-resolution regional map of endocardial strain can be computed as SQUEEZ − 1 at each point (Figure 1). This metric has been correlated with circumferential strain (E_cc_), the criterion standard for noninvasive regional strain, using tagged CMR sequences in a canine model of myocardial infarction.^14^

**Figure 2:**
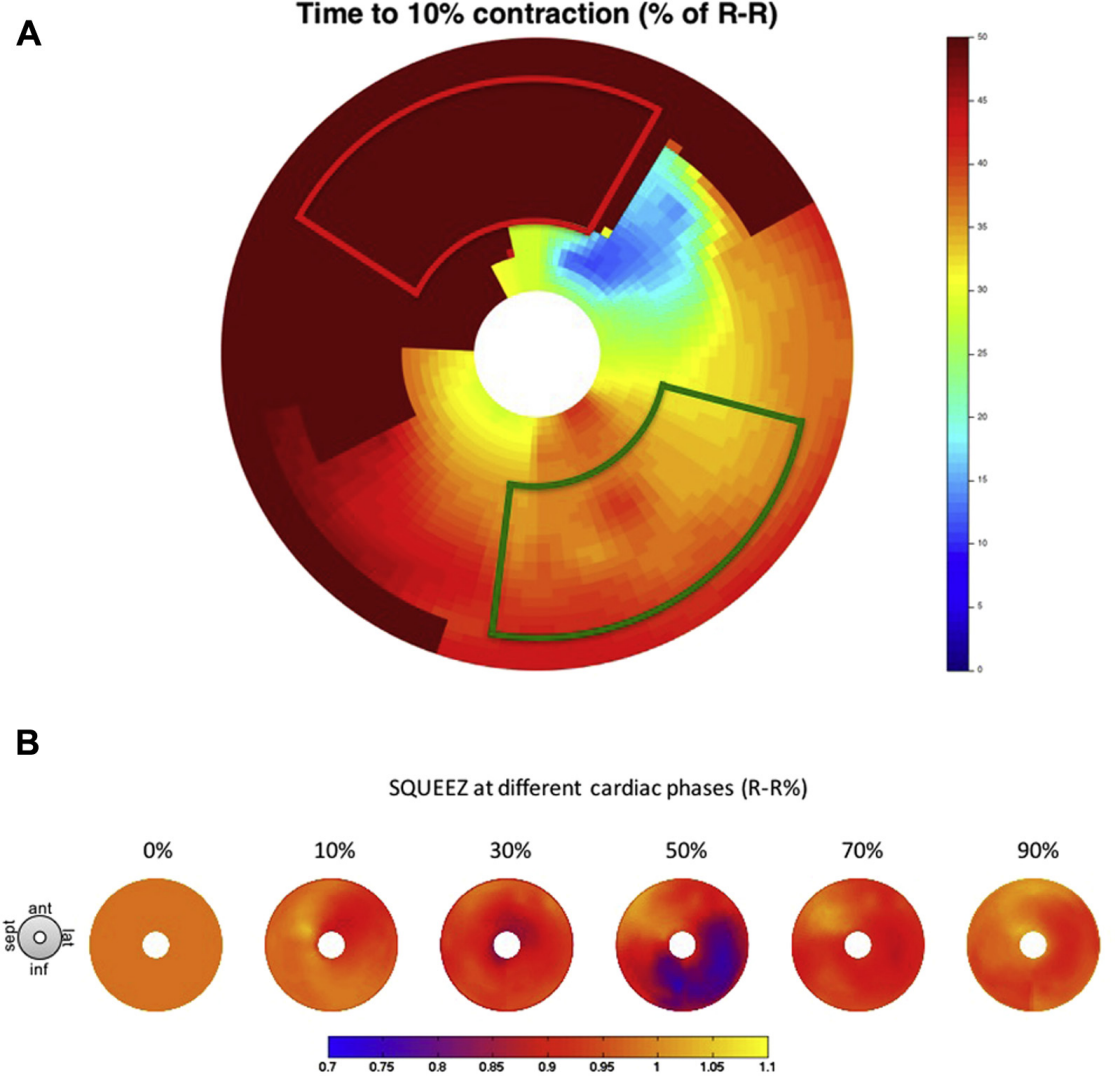
**A:** Bull’s-eye plot of the time delay (color scale, in milliseconds) until 10% shortening occurs (ie, time for SQUEEZ value to reduce from 1.0 to 0.9) across left ventricular regions. Dark red/brown shows anterior and anteroseptal segments not achieving 10% shortening and likely represents infarcted myocardium. The outlined red box represents areas to avoid (akinetic segments). Red-colored regions in the inferolateral wall show the latest activation away from areas of scar; the outlined green box shows the target pacing regions. **B:** Bull’s-eye plot with color scale representing SQUEEZ values. All segments begin at a SQUEEZ value of 1. Yellow represents a SQUEEZ value of >1 (paradoxical stretch/dyskinesis in septal regions). Blue represents a SQUEEZ value of <0.8, and viable regions with reasonable shortening deemed as good targets. SQUEEZ = stretch quantifier for endocardial engraved zones.

SQUEEZ-derived regional function data were merged with individual patient anatomy. Septal segments were excluded as targets. SQUEEZ-derived strain curves with low amplitude strain (LAS) <10% shortening were judged nonviable and excluded as targets (analogous to echocardiographic data with poor CRT response with LV lead placement in segments with LAS <9.8%).^11^, ^12^ Regional time to peak strain (minimal SQUEEZ value) was calculated using the individual heart rate/cycle length for the CT acquisition (Figure 2). Given the distribution of the coronary veins, the time to peak strain of myocardial segments overlying the same coronary vein was averaged to produce anterior, lateral, and inferior values for mechanical activation delay and compared with hemodynamic data.

### CRT implantation

CRT upgrade was performed with a quadripolar LV lead in 17 patients (94%) and a bipolar lead in 1 because of unfavorable venous anatomy. Nine patients (50%) received CRT with defibrillator; 1 patient required an additional RV shock lead; and the remaining 8 already having preexisting implantable cardioverter-defibrillators. Identification of the optimal hemodynamic site for LV stimulation was performed by measuring acute hemodynamic response (AHR) using an 0.014-in high-fidelity Certus RADI PressureWire (St. Jude Medical, St. Paul, MN) in the LV cavity via a retrograde arterial approach as previously described.^13^ Atrial pacing 10 beats/min above the intrinsic rate or RV pacing (DDDRV) for patients with no underlying rhythm was baseline. Atrioventricular delays were fixed at 100 ms and ventriculoventricular delay at 0 ms. AHR for each venous site compared biventricular pacing with baseline (% change, dP/dt_max,_ mm Hg/s). AHR was compared with CT indices and the sensed QLV interval at each pacing site.^14^ Patients underwent 6-month follow-up to identify clinical responders via the Packer score^15^ and echocardiographic response defined as reduction in LV end-systolic volume >15%. The primary end point was cardiac CT–derived regional endocardial strain analysis (SQUEEZ) prediction of the pacing site achieving the optimal AHR.

### Statistics

Continuous data were presented as mean ± SD. Those with Gaussian distribution were compared using a paired *t* test; those with a non-Gaussian distribution were compared using the Wilcoxon matched-pair signed rank test. Categorical data were presented as absolute number of occurrences and associated frequency (%). Analysis of variance was used to compare >2 groups. The results were considered significant at a *P* value of <.05.

## Results

The patient demographic characteristics are summarized in Table 1. Patients had a high percentage of RV pacing with a mean QRS duration of 173 ± 21 ms. Eight patients (44%) had ICM.

**Table 1:** Demographic characteristics

Characteristic	Value
Age (y)	68.8 ± 15.5
Sex: male	14 (78)
Ischemic cardiomyopathy	8 (44)
RV pacing (%) pre-CRT	92.2 ± 17
LBBB	15 (83)
QRS duration (ms)	173 ± 21
Sinus rhythm	16 (89)
LV end-diastolic volume (mL)	186 ± 65
LV end-systolic volume (mL)	128 ± 62
LV ejection fraction (%)	34 ± 10

Values are presented as mean ± SD or as n (%).

CRT = cardiac resynchronization therapy; LBBB = left bundle branch block; LV = left ventricular; RV = right ventricular.

### Quality of CT data sets

All CT scans were successfully completed (mean heart rate 64 ± 7 beats/min; mean radiation dose-area product 1194 ± 419 mGy·cm^2^). Patients were supine for 15 ± 1 minutes. Studies were independently assessed using the Society of Cardiovascular Computed Tomography quality score^16^ (mean score 3.4 ± 1.3 out of 5). The main reason for reduced scoring was beam hardening artifact from existing pacing wires. Wall thinning/hypoperfusion (inferring scar) was present in 7 of 8 patients with ICM; however, late contrast enhancement was limited to 1 patient.

### CT-SQUEEZ–derived target segments to predict the optimal venous target

**Figure 3:**
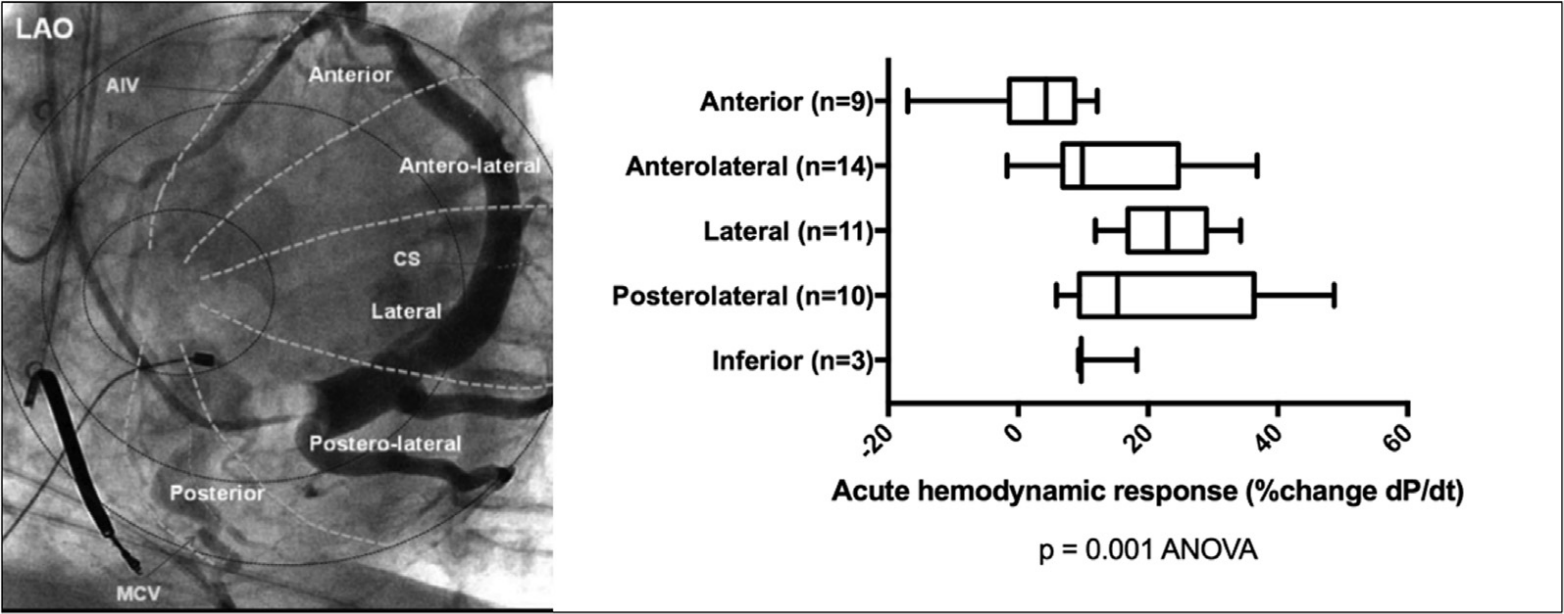
*Left:* Occlusive venography with nomenclature for the coronary venous tree. Reproduced with permission from Spencer et al.^17^*Right:* Regional acute hemodynamic response by coronary vein tested. Box-and-whisker plot for each vein detailing the mean (solid line), range, and SD. Acute hemodynamic response values are % change in dP/dt vs baseline. There was a significant difference between groups: *P* = .001 (ANOVA). AIV = anterior interventricular vein; ANOVA = analysis of variance; CS = coronary sinus; LAO = left anterior oblique; MCV = middle cardiac vein.

Eighteen patients underwent successful LV lead implantation, and 15 of 18 had full hemodynamic and CT data sets. In 1 patient a RADI wire was not sited because of arterial access, and 2 patients had CT scans of insufficient quality for analysis. CT-SQUEEZ analysis identifying the target epicardial vein subtending the area of LMA excluding LAS regions (inferring scar) was compared with all sites where AHR was measured (3 ± 1 coronary veins per patient). AHR (mean % change) was as follows: 2.5% ± 8.8%, anterior; 14.5% ± 11.5%, anterolateral; 23.2% ± 7.7%, lateral; 21.8% ± 15.0%, posterolateral; and 12.4% ± 5.1%, posterior (analysis of variance, *P* = .001). Lateral and posterolateral veins produced the best AHR irrespective of etiology. The lateral vein stimulation produced greater AHR than did the anterior vein (23.2% ± 8.8% vs 2.5% ± 8.8%; *P* < .001) (Figure 3). Notably 2 of 15 patients (13%) had no epicardial vein supplying the CT target. In the remaining 13, the CT-SQUEEZ target determined LV implantation site, achieving maximal AHR in 9 (70%). A >10% increase in dP/dt_max_ (positive AHR)^13^ was achieved in 22% anterior, 50% anterolateral, 100% lateral, 80% posterolateral, and 67% posterior sites tested. AHR >10% was achieved in 12 of 13 patients (92%) with an epicardial vein supplying a CT-SQUEEZ target (the remaining patient had a target AHR of 6.8%).

### CT vs hemodynamic/electrical guidance (Figure 4)

CT-SQUEEZ targets produced AHR similar to the best achievable AHR (20.4% ± 13.7% vs 24.9% ± 11.1%; *P* = .36). Targeting electrical latency (longest QLV interval) achieved a similar AHR (19.4% ± 11.5%; *P* = .85). Pacing scar produced the lowest AHR (6.8% ± 3.2%; *P* = .04 vs CT) comparable to the worst achievable AHR (6.4% ± 3.1%; *P* = .01 vs CT). AHR and QLV interval weakly correlated in 63 paired data sets (Pearson *r* = 0.31; *P* = .01). The AHR of locations with QLV interval >100 ms but in scar was significantly lower than that of nonscarred locations (5.2% ± 1.5% vs 19.5% ± 9.4%; *P* = .005) (Figure 5).

**Figure 4:**
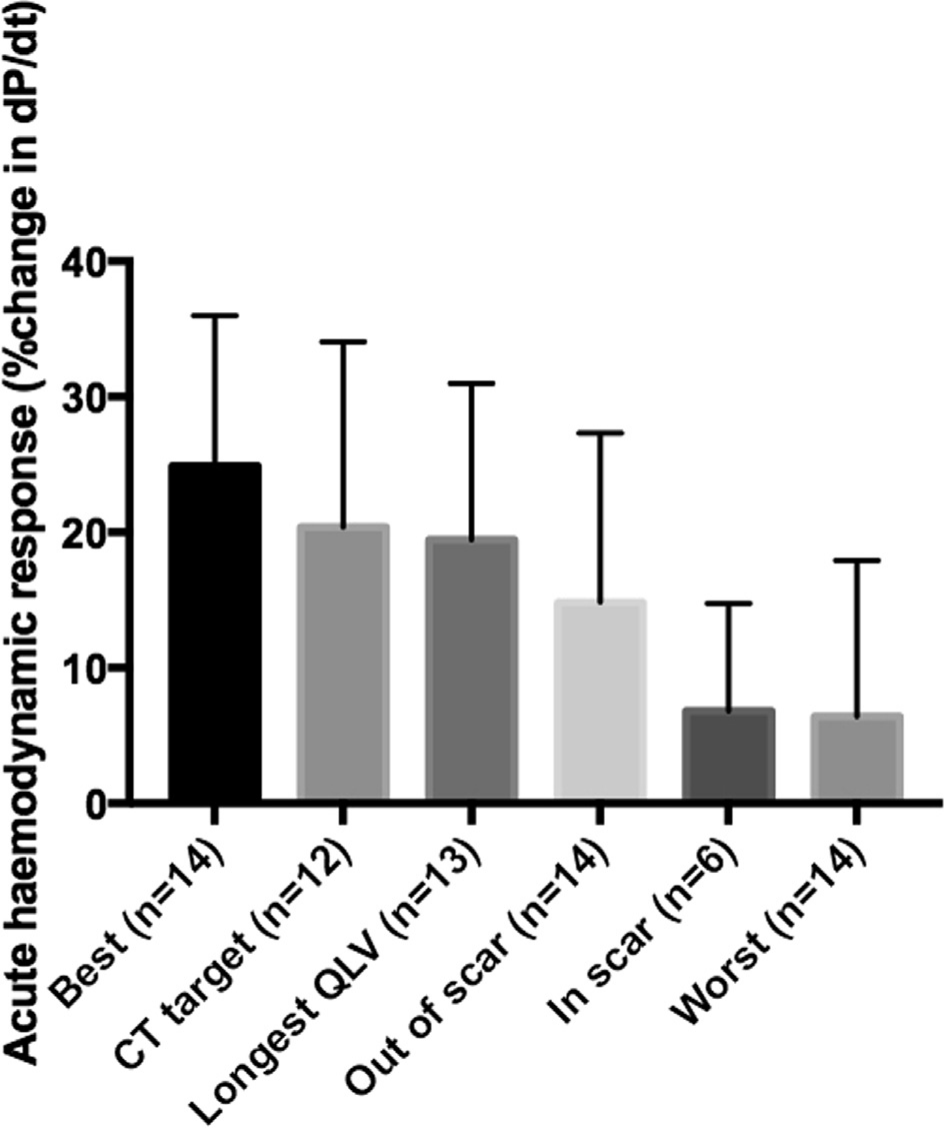
Percentage change AHR determined by pacing the vein with optimal AHR per patient (Best, n = 14), the CT-SQUEEZ–derived target (CT target, n = 12), greatest electrical latency (Longest QLV, n = 13), absence of scar (Out of scar, n = 14), presence of scar (In scar, n = 6), and the vein with the worst AHR per patient (Worst, n = 14). Best vs CT target, *P* = .36; Best vs Longest QLV, *P* = .22; Best vs Out of scar, *P* = .03; Best vs In scar, *P* = .002; Best vs Worst, *P* = .0002; CT target vs Longest QLV, *P* = .85; CT target vs Out of scar, *P* = .29; CT target vs In scar, *P* = .04; CT target vs Worst, *P* = .009. AHR = acute hemodynamic response; CT = computed tomography; SQUEEZ = stretch quantifier for endocardial engraved zones.

**Figure 5:**
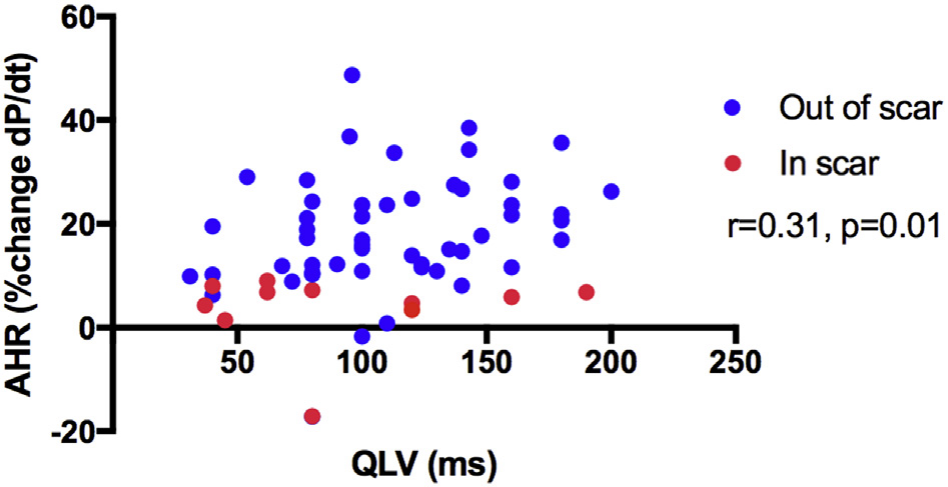
Scatterplot of AHR vs QLV interval. Each patient had multiple data points acquired. There is a weak correlation between AHR and QLV interval (*r* = 0.31; *P* = .01). Locations in scar (red) had a lower AHR than did locations out of scar (blue). AHR = acute hemodynamic response.

### CRT response (Table 2)

**Figure 6:**
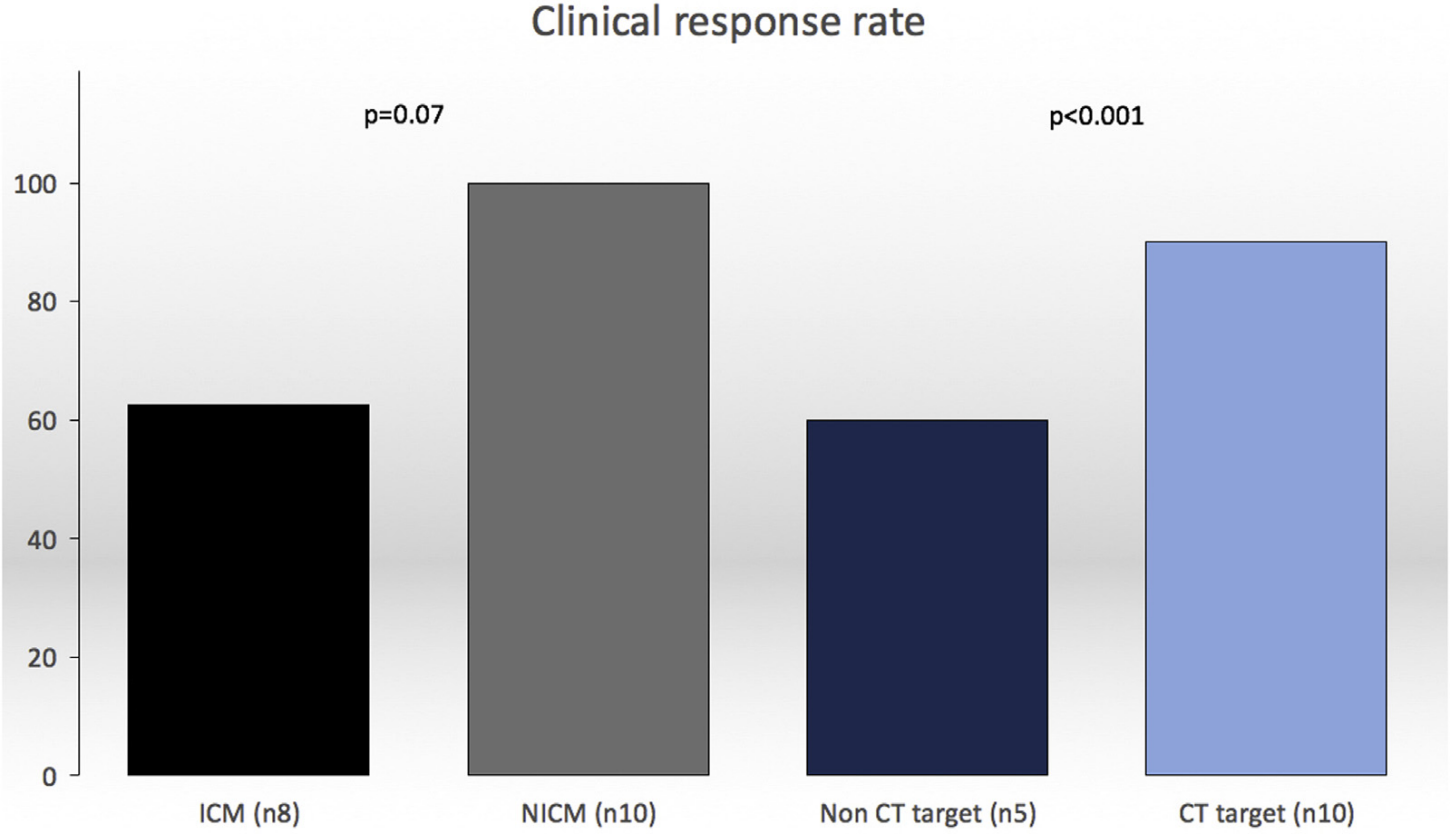
Clinical response rates in CT-SQUEEZ target (n = 10) vs nontarget (n = 5) (*P* < .001) and ICM (n = 8) vs NICM (n = 10) (*P* = .07). CT = computed tomography; ICM = ischemic cardiomyopathy; NICM = nonischemic cardiomyopathy; SQUEEZ = stretch quantifier for endocardial engraved zones.

At 6 months, patients symptomatically improved (New York Heart Association 1.7 ± 0.7 vs 2.8 ± 0.4; *P* < .001 and Minnesota Living with Heart Failure questionnaire scores 32 ± 24 vs 39 ± 19; *P* = .03) and were able to walk on average 92 m further over 6 minutes. The paced QRS duration was shorter (142 ± 18 ms vs 173 ± 21 ms; *P* < .001), LV ejection fraction increased (34% ± 10% to 44% ± 15%; *P* = .001), and N-terminal pro-brain natriuretic peptide level decreased (472 ± 459 vs 1121 ± 749; *P* = .003). Patients paced in CT-SQUEEZ targets had greater clinical response than did those paced in nontarget segments (90% vs 60%; *P* < .001) (Figure 6). Hundred percent of patients with non-ICM were clinical and echocardiographic responders as compared with only 63% of patients with ICM (*P* = .07).

**Table 2:** CRT response

Variable	Preassessment	6-mo follow-up	*P*
NYHA class symptoms	2.8 ± 0.4	1.7 ± 0.7	<.001
MLHF questionnaire points	39 ± 19	32 ± 24	.03
6-minute walking distance (m)	291 ± 137	383 ± 154	.06
Paced QRS duration (ms)	173 ± 21	142 ± 18	<.001
CPET, Vo_2_max (mL/(min·kg))	17.9 ± 4	18.1 ± 5	.84
CPET, slope @ Vo_2_max	36 ± 9	34 ± 5	.88
LV end-diastolic volume (mL)	186 ± 65	154 ± 61	.01
LV end-systolic volume (mL)	128 ± 62	93 ± 60	.01
2D LVEF (TTE) (%)	34 ± 10	44 ± 15	.001
NT-pro-BNP level	1121 ± 749	472 ± 459	.003

Values are presented as mean ± SD.

2D = 2-dimensional; CPET = cardiopulmonary exercise test; CRT = cardiac resynchronization therapy; LV = left ventricular; LVEF = left ventricular ejection fraction; MLHF = Minnesota Living with Heart Failure questionnaire; NT-pro-BNP = N-terminal pro-brain natriuretic peptide; NYHA = New York Heart Association; TTE = transthoracic echocardiogram; Vo_2_max = maximum oxygen consumption.

## Discussion

The principal findings were as follows:

1. CT-SQUEEZ targets produced a mean AHR similar to the best achievable AHR (20.4% ± 13.7% vs 24.9% ± 11.1%; *P* = .36).
2. CT-SQUEEZ guidance produced positive AHR in 92% of cases when a target segment was paced.
3. Pacing a CT-SQUEEZ target vein produced greater clinical response vs nontarget segments (90% vs 60%).

We demonstrate the novel utility of preprocedural CT for LV lead guidance to regions of LMA devoid of LAS. Figures 1 and 2 show that cardiac CT sequences were able to generate functional data sets with sufficient temporal resolution to differentiate the region of LMA. In addition to wall thinning and hypoperfusion, the SQUEEZ algorithm inferred regions of scar on the basis of LAS in keeping with echocardiographic studies (defined LAS as radial strain <10%), resulting in suboptimal response to CRT.^15^ Our CT protocol included a late enhancement sequence to identify fibrosis; however, we identified only late enhancement in 1 patient. Our CT-SQUEEZ–derived algorithm predicted regions with an AHR within 2.5% of the maximum in 11 of 12 cases (92%). In addition, patients paced in CT targets had a more favorable response (Figure 4). In 1 case, the mismatch between the CT target and the vein with the best AHR was significant (22.2%). In 2 cases, CT-SQUEEZ targeted the posterior wall; however, the patients had no epicardial vein overlying this site.

The clinical utility of AHR >10% in predicting CRT response has been demonstrated,^16^ and using this cutoff, a high proportion of CT targets had a positive AHR. There was a trend toward greater AHR in sites out of scar vs CT inferred scar (14.8% ± 12% vs 6.8% ± 8%; *P* = .17). The mean AHR was similar in CT targets compared with the QLV interval (Figure 4), reflecting a correlation between electrical and mechanical dyssynchrony. However, regions with electrical latency (QLV interval >100 ms) in scar resulted in suboptimal AHR, suggesting that the QLV interval alone may fail to identify the optimal stimulation site in the presence of scar.

We previously demonstrated that CMR-derived scar and dyssynchrony can guide LV lead implantation^18^ with excellent CRT response if pacing in a CMR target in keeping with the present findings. Similarly, Laksman et al^19^ used CMR-derived scar and dyssynchrony-guided lead placement with echocardiographic super-response in 58% of patients. Currently there are limited clinical data describing the use of cardiac CT LV lead guidance.^20^ Two randomized controlled studies TARGET^21^ and STARTER^22^ have shown benefit with LV lead guidance using speckle tracking echocardiography.

Scar avoidance using CMR-derived late gadolinium enhancement is associated with improved cardiovascular outcomes.^23^ The ability of CMR to accurately define scar is superior to CT; however, almost one-third of patients undergoing CRT have existing pacemakers unsuitable for CMR.^5^ Cardiac CT offers several potential advantages over CMR. Images of superior spatial resolution within a 3-dimensional isotropic data set are acquired within a matter of seconds; the new generation of CT scanners produce images in a single heartbeat. In addition, an exquisite differentiation of endocardium and blood pool enables accurate tracking of regional surfaces over the duration of the cardiac cycle. Delayed triggered contrast-enhanced sequences facilitate good opacification of the coronary venous tree, and volume-rendered reconstructions can delineate the course of potential venous targets before implantation. This may be particularly advantageous as coronary venous anatomy is highly variable and may impact successful CRT delivery.^24^ Furthermore, identification of coronary sinus valves and highly angulated/tortuous vessels may help in patients with a previously failed implant. While targeting regions of LMA is scientifically sound, LV lead placement is restricted by coronary vein anatomy that may not always overly the optimal region. In a cardiac CT study of 121 postmortem hearts, 29% had no coronary vein overlying the posterolateral region.^17^ Lack of a venous target identified by preprocedural CT may allow alternative forms of LV stimulation (multisite/endocardial) to be considered.^25^, ^26^

### Study limitations

Modern single energy source CT scanners are currently unable to accurately delineate extracellular myocardial fibrosis through late contrast enhancement. While iodinated contrast displays similar kinetic properties to gadolinium-DTPA (diethylenetriamine penta-acetic acid) and can demonstrate acute hypoperfusion from ischemic injury, reliable visualization of chronic fibrosis with late enhancement occurred in only 1 patient. We therefore used local wall thinning (<6 mm) and/or hypoperfusion to infer scar, and the low AHR in these segments is in keeping with scar despite the lack of visualization (Figures 4 and 5). The presence of existing pacing systems resulting in beam hardening artifact and degradation of signal in myocardial tissue local to the pacing leads may explain this. One study reporting a good correlation between late enhancement and scar in histological macroscopy in a chronic porcine mode^25^ used higher-contrast doses than our study (145 ± 35 mL vs 120 ± 0 mL; *P* = .005), and the animals had no preexisting pacing systems. The development of dual energy source CT scanners holds promise in improving differentiation between subtle soft tissue characteristics and may be able to more reliably demonstrate myocardial fibrosis.^27^ The temporal resolution of cardiac CT in this study (70–100 ms) is inferior to echocardiography (20 ms) and CMR (35–50 ms, obtained over multiple beats), and cardiac CT may be less sensitive to subtle regional motion changes. While the SQUEEZ algorithm detects motion abnormalities with high resolution, only lower-resolution estimates (16 standard AHA segments) were needed. Furthermore, the CT-SQUEEZ–derived metric has been shown to correlate well with circumferential strain (E_cc_) in an animal model, suggesting that it is sensitive enough to demonstrate local regional motion differences and remain a useful tool to assess dyssynchrony.^14^ The utility of AHR in predicting CRT response^16^ is limited, and the results of a large multicenter randomized trial of AHR are awaited (RADI-CRT, Clinical Trial Registration No.: NCT01464502).

## Conclusion

Preprocedural CT-SQUEEZ–derived target selection may be a valuable tool to predict the optimal venous site for LV lead placement by guiding the implanter toward late activating regions, away from areas of scar.

## References

[1] Curtis A.B., Worley S.J., Adamson P.B., Chung E.S., Niazi I., Sherfesee L., Shinn T., Sutton M.S.J. (2013). Biventricular pacing for atrioventricular block and systolic dysfunction. N Engl J Med.

[2] Bleeker G.B., Kaandorp T.A., Lamb H.J., Boersma E., Steendijk P., de Roos A., Van Der Wall E.E., Schalij M.J., Bax J.J. (2006). Effect of posterolateral scar tissue on clinical and echocardiographic improvement after cardiac resynchronization therapy. Circulation.

[3] Ypenburg C., van Bommel R.J., Delgado V., Mollema S.A., Bleeker G.B., Boersma E., Schalij M.J., Bax J.J. (2008). Optimal left ventricular lead position predicts reverse remodeling and survival after cardiac resynchronization therapy. J Am Coll Cardiol.

[4] Leyva F. (2010). Cardiac resynchronization therapy guided by cardiovascular magnetic resonance. J Cardiovasc Magn Reson.

[5] Daubert J.-C., Saxon L., Adamson P.B. (2012). 2012 EHRA/HRS expert consensus statement on cardiac resynchronization therapy in heart failure: implant and follow-up recommendations and management. Europace.

[6] Mak G.S., Truong Q.A. (2012). Cardiac CT: imaging of and through cardiac devices. Curr Cardiovasc Imaging Rep.

[7] Sun C., Pan Y., Wang H., Li J., Nie P., Wang X., Ma H., Huo F. (2014). Assessment of the coronary venous system using 256-slice computed tomography. PLoS One.

[8] Truong Q.A., Singh J.P., Cannon C.P. (2008). Quantitative analysis of intraventricular dyssynchrony using wall thickness by multidetector computed tomography. JACC Cardiovasc Imaging.

[9] Truong Q.A., Thai W., Wai B. (2014). Myocardial scar imaging by standard single-energy and dual-energy late enhancement CT: comparison with pathology and electroanatomic map in an experimental chronic infarct porcine model. J Cardiovasc Comput Tomogr.

[10] Pourmorteza A., Schuleri K.H., Herzka D.A., Lardo A.C., McVeigh E.R. (2012). A new method for cardiac computed tomography regional function assessment: stretch quantifier for endocardial engraved zones (SQUEEZ). Circ Cardiovasc Imaging.

[11] Mendoza D.D., Joshi S.B., Weissman G., Taylor A.J., Weigold W.G. (2010). Viability imaging by cardiac computed tomography. J Cardiovasc Comput Tomogr.

[12] Nieman K., Cury R.C., Ferencik M., Nomura C.H., Abbara S., Hoffmann U., Gold H.K., Jang I.-K., Brady T.J. (2006). Differentiation of recent and chronic myocardial infarction by cardiac computed tomography. Am J Cardiol.

[13] Blankstein R., Rogers I.S., Cury R.C. (2009). Practical tips and tricks in cardiovascular computed tomography: diagnosis of myocardial infarction. J Cardiovasc Comput Tomogr.

[14] Pourmorteza A., Chen M.Y., Pals J., Arai A.E., Mcveigh E.R. (2016). Correlation of CT-based regional cardiac function (SQUEEZ) with myocardial strain calculated from tagged MRI: an experimental study. Int J Cardiovasc Imaging.

[15] Khan F.Z., Virdee M.S., Read P.A. (2010). Effect of low-amplitude two-dimensional radial strain at left ventricular pacing sites on response to cardiac resynchronization therapy. J Am Soc Echocardiogr.

[16] Duckett S.G., Ginks M.R., Shetty A.K., Bostock J., Gill J.S., Hamid S., Kapetanakis S., Cunliffe E., Razavi R., Carr-White G., Rinaldi C.A. (2011). Invasive acute hemodynamic response to guide left ventricular lead implantation predicts chronic remodeling in patients undergoing cardiac resynchronization therapy. J Am Coll Cardiol.

[17] Spencer J.H., Larson A.A., Drake R., Iaizzo P.A. (2014). A detailed assessment of the human coronary venous system using contrast computed tomography of perfusion-fixed specimens. Heart Rhythm.

[18] Shetty A.K., Duckett S.G., Ginks M.R. (2013). Cardiac magnetic resonance-derived anatomy, scar, and dyssynchrony fused with fluoroscopy to guide LV lead placement in cardiac resynchronization therapy: a comparison with acute haemodynamic measures and echocardiographic reverse remodelling. Eur Heart J Cardiovasc Imaging.

[19] Laksman Z., Yee R., Stirrat J. (2014). Model-based navigation of left and right ventricular leads to optimal targets for cardiac resynchronization therapy: a single centre feasibility study. Circ Arrhythm Electrophysiol.

[20] Zhou W., Hou X., Piccinelli M., Tang X., Tang L., Cao K., Garcia E.V., Zou J., Chen J. (2014). 3D fusion of LV venous anatomy on fluoroscopy venograms with epicardial surface on SPECT myocardial perfusion images for guiding CRT LV lead placement. JACC Cardiovasc Imaging.

[21] Khan F.Z., Virdee M.S., Palmer C.R., Pugh P.J., O’Halloran D., Elsik M., Read P.A., Begley D., Fynn S.P., Dutka D.P. (2012). Targeted left ventricular lead placement to guide cardiac resynchronization therapy: the TARGET study: a randomized, controlled trial. J Am Coll Cardiol.

[22] Saba S., Marek J., Schwartzman D., Jain S., Adelstein E., White P., Oyenuga O.A., Onishi T., Soman P., Gorcsan J. (2013). Echocardiography-guided left ventricular lead placement for cardiac resynchronization therapy: results of the Speckle Tracking Assisted Resynchronization Therapy for Electrode Region trial. Circ Heart Fail.

[23] Leyva F., Foley P.W.X., Chalil S., Ratib K., Smith R.E., Prinzen F., Auricchio A. (2011). Cardiac resynchronization therapy guided by late gadolinium-enhancement cardiovascular magnetic resonance. J Cardiovasc Magn Reson.

[24] Singh J.P., Houser S., Heist E.K., Ruskin J.N. (2005). The coronary venous anatomy. J Am Coll Cardiol.

[25] Rinaldi C.A., Burri H., Thibault B., Curnis A., Rao A., Gras D., Sperzel J., Singh J.P., Biffi M., Bordachar P., Leclercq C. (2015). A review of multisite pacing to achieve cardiac resynchronization therapy. Europace.

[26] Behar J.M., Jackson T., Hyde E., Claridge S., Gill J., Bostock J., Sohal M., Porter B., O’Neill M., Razavi R., Niederer S., Rinaldi C.A. (2016). Optimized left ventricular endocardial stimulation is superior to optimized epicardial stimulation in ischemic patients with poor response to cardiac resynchronization therapy. JACC Clin Electrophysiol.

[27] Hamilton-Craig C., Seltmann M., Ropers D., Achenbach S. (2011). Myocardial viability by dual-energy delayed enhancement computed tomography. JACC Cardiovasc Imaging.

