# Supplementary material for: Comprehensive use of cardiac computed tomography to guide left ventricular lead placement in cardiac resynchronization therapy
Source: Heart Rhythm. 2017 Sep;14(9):1364–72. doi: 10.1016/j.hrthm.2017.04.041 (PMC5575356; doi:10.1016/j.hrthm.2017.04.041)
Supplement: Supplemental Data [file mmc1.docx]

**Supplemental data 1**. Target LV region by CT-SQUEEZ (aiming for latest mechanical activation, LMA avoiding areas of low amplitude strain, LAS) and by AHR. nd=no data RADI=pressure wire in the LV

| Case | Target region by CT-SQUEEZ (LMA, avoiding LAS) | Target vein by AHR | CT-SQUEEZ to AHR concordance | Comments |
| --- | --- | --- | --- | --- |
| 1 | Inferior | Inferior | Y |  |
| 2 | Inferior | Anterolateral | N | Only venous option, adjacent to anterior scar |
| 3 | Inferior | Lateral | N |  |
| 4 | CT nd. | Inferior | - |  |
| 5 | Inferior | Lateral | N |  |
| 6 | Lateral | Lateral | Y |  |
| 7 | Lateral | Lateral | Y |  |
| 8 | Lateral | Lateral | Y | Anterior LMA but in scar, therefore Lateral chosen |
| 9 | Inferior | Anterolateral | Y | Only venous option |
| 10 | Anterior | Inferolateral | N |  |
| 11 | Lateral or Inferior | Inferolateral | Y | Lateral and Inferior regions - same LMA |
| 12 | Lateral | Lateral | Y | Anterior LMA but in scar, therefore Lateral chosen |
| 13 | Inferior | RADI nd | - |  |
| 14 | CT nd. | Anterolateral | - |  |
| 15 | Inferolateral | Inferolateral | Y |  |
| 16 | Lateral | Lateral | Y |  |
| 17 | Inferolateral | Inferolateral | Y | Anterior LMA but in scar, therefore Inferolateral chosen |
| 18 | Lateral | Lateral | Y |  |
